# The Effects of a 3-Week Heartbeat Perception Training on Interoceptive Abilities

**DOI:** 10.3389/fnins.2022.838055

**Published:** 2022-05-09

**Authors:** Christine Schillings, Georgios Karanassios, Niklas Schulte, Dana Schultchen, Olga Pollatos

**Affiliations:** ^1^Department of Clinical and Health Psychology, Faculty of Engineering, Computer Science and Psychology, Institute of Psychology and Education, University of Ulm, Ulm, Germany; ^2^Department of Work and Organizational Psychology, Faculty of Engineering, Compurter Science and Psychology, Institute of Psychology and Education, University of Ulm, Ulm, Germany

**Keywords:** heartbeat perception training, interoception, cardiac interoceptive accuracy, interoceptive sensibility, interoceptive training

## Abstract

Recent studies showed promising short-term effects of heartbeat perception training on interoceptive abilities. Research on the effects of heartbeat perception training on interoceptive abilities over time is sparse. Therefore, the aim of this study was to examine the short-term effects and the effects of a 3-week heartbeat perception training over time on interoceptive abilities, namely, cardiac interoceptive accuracy (IAc) and interoceptive sensibility (IS). A total of 40 healthy participants were randomized to the intervention group (*n* = 20) or the control group (*n* = 20). The intervention group conducted three cardiac biofeedback sessions (one per week) at the laboratory, whereas the control group watched a documentary instead. Interoceptive abilities were assessed *via* the heartbeat perception task (IAc) and confidence ratings (IS) at baseline, after each laboratory session, and 1 week after the last session (post-measurement). IAc was significantly increased in the intervention group compared to the control group after the first training session (short-term effect). There were no significant improvements in IS due to the first session, and neither on IAc nor IS over time. Descriptive trends of improved interoceptive abilities over time were found in both groups. Single session of heartbeat perception training seems to be a promising approach to improve IAc. Future research should further investigate the long-term effects of diverse heartbeat perception training varying in frequency and intensity of the training sessions in diverse samples aiming to improve interoceptive abilities.

## Introduction

Interoception describes the process by which the nervous system senses, interprets, and integrates signals from the internal body aiming at a moment-by-moment internal bodily landscape across conscious and unconscious levels (Khalsa et al., 2018). Corresponding to the three-dimensional model of interoception developed by Garfinkel et al. (2015), interoceptive accuracy, i.e., cardiac interoceptive accuracy (IAc), is defined as the objective performance in detecting interoceptive (cardiac) signals. It can be assessed *via* performance tasks, such as the heartbeat counting task (Schandry, 1981) or the heartbeat discrimination task (Whitehead et al., 1977). Interoceptive sensibility (IS) comprises the subjective beliefs about the own ability to focus on internal bodily sensations, quantified by self-report measures, such as the Body Perception Questionnaire (Porges, 1993, 2015), the Interoceptive Accuracy Scale (Murphy et al., 2020), or confidence ratings concerning IAc (Garfinkel et al., 2015).

In previous research, interoception emerged as a health-related variable. Exemplarily, high interoceptive abilities were shown to be related to more intense perception and better regulation of emotions (e.g., Barrett et al., 2004; Herbert and Pollatos, 2008; Dunn et al., 2010; Füstös et al., 2013; Zamariola et al., 2019a) and higher empathy (Grynberg and Pollatos, 2015). However, Zamariola et al. (2019b) found that interoceptive abilities did not modulate negative affect. In contrast, impaired interoceptive abilities were found in various mental disorders, such as anorexia nervosa (Pollatos et al., 2008; Fischer et al., 2016), depression (Pollatos et al., 2009; Eggart et al., 2019; Schultchen et al., 2021), somatoform disorders (Weiss et al., 2014), obsessive-compulsive disorders (Schultchen et al., 2019b), or schizophrenia (Koreki et al., 2020). However, it needs to be noted that evidence concerning the association between interoceptive abilities and somatic symptoms and related disorders (e.g., Mussgay et al., 1999; Scholz et al., 2001) as well as regarding anxiety or panic disorders (e.g., Domschke et al., 2010; Stevens et al., 2011; Krautwurst et al., 2016) is mixed. These inconsistent findings might result from methodological differences between the studies, such as various instructions. Moreover, associations between interoceptive abilities and stress were found (e.g., Schulz and Vögele, 2015; Maeda et al., 2019; Schultchen et al., 2019a; Schulz et al., 2020; Opdensteinen et al., 2021). Furthermore, decreased interoceptive abilities have been assumed to play a role in the perception of somatic symptoms (van den Bergh et al., 2017, 2019). Consequently, investigating different approaches to improve interoceptive abilities is of high relevance.

A growing body of studies showed increased interoceptive abilities due to diverse interventions of various lengths, such as self-focused procedures (Weisz et al., 1988; Tsakiris et al., 2011; Ainley et al., 2012; Ainley and Tsakiris, 2013; Pollatos et al., 2016), mindfulness-based interventions (e.g., Bornemann et al., 2014; Parkin et al., 2014; Bornemann and Singer, 2017; Fischer et al., 2017), floating (Feinstein et al., 2018), power posing (Weineck et al., 2019), or cognitive behavioral therapy in depressive patients (Karanassios et al., 2021). Concerning the effects of mindfulness-based interventions on interoceptive abilities, previous findings are incongruent. For example, Fischer et al. (2017) found improvements in IAc but no changes in IS due to an 8-week body scan intervention. In contrast, short-term mindfulness-based interventions, such as a single yoga session (Schillings et al., 2021) or two meditation sessions (Fairclough and Goodwin, 2007), had no significant effect on IAc. Consequently, further research to investigate effective interventions aiming to improve interoceptive abilities that also include repeated intervention sessions might be necessary.

First studies (Schandry and Weitkunat, 1990; Schaefer et al., 2014; Meyerholz et al., 2019) showed increased interoceptive abilities due to heartbeat perception training. In an early study by Schandry and Weitkunat (1990) investigating a cardiac awareness training, participants were instructed to press a button after every perceived heartbeat over several training blocks. For correct responses, they received acoustic feedback. In two training groups (i.e., consistent-tone and fading-tone groups), IAc, as measured *via* the heartbeat discrimination task (Whitehead et al., 1977), increased. Meyerholz et al. (2019) reported higher IAc in healthy participants who conducted a contingent heartbeat perception training compared to a non-contingent feedback condition, a mindfulness practice, and a waiting condition. This heartbeat perception training paradigm developed by Schaefer et al. (2014) consisted of an interoceptive learning task concerning cardiac perception, including phases with and without feedback on individual performance. Similarly, Schenk et al. (2020) showed an increase in IAc in healthy participants after a single session of the heartbeat perception training paradigm in a stress condition (cold pressor stress test). Contrarily, in the study conducted by Schaefer et al. (2014), the same training showed no effect as compared to a waiting control group for a sample of patients with somatoform disorders, i.e., a population with low interoceptive abilities (Weiss et al., 2014). In contrast to the other aforementioned studies, Rominger et al. (2021) used the heartbeat discrimination task (Whitehead et al., 1977) to assess IAc, which might account for the heterogeneous results. To conclude, there is first but also mixed evidence that heartbeat perception training improves individual cardiac perception (Schandry and Weitkunat, 1990; Meyerholz et al., 2019; Schenk et al., 2020).

Referring to more common cardiac trainings, e.g., biofeedback which is based on heart rate (e.g., Peira et al., 2013, 2014) or heart rate variability (e.g., Kotozaki et al., 2014; Goessl et al., 2017), positive changes in health-related variables were found. In particular, previous research has showed improvements in clinical symptoms in several physical and mental disorders, such as cardiovascular diseases, chronic obstructive pulmonary disease, fibromyalgia, major depressive disorder, and post-traumatic stress disorders (for a review, refer to Wheat and Larkin, 2010), cardiac control in emotional reactions, i.e., emotion regulation (Peira et al., 2013, 2014), and increases and decreases in baroreceptor functions. A meta-analysis proposed by Goessl et al. (2017) on the basis of healthy and clinical samples’ reports reduced stress levels due to biofeedback based on heart rate variability. It needs to be noted that these studies differ in their lengths of the implemented biofeedback training interventions and in their number of sessions. In contrast, e.g., Peira et al. (2013, 2014) examined the short-term effects of a single biofeedback session, Del Paso and González (2004) applied a 3-week biofeedback training targeting the baroreceptor sensitivity with one session per week, and Kotozaki et al. (2014), as one of the studies included in the meta-analysis by Goessl et al. (2017), showed decreased stress levels due to a 4-week biofeedback intervention with daily sessions.

Consequently, differentiating between short- and long-term effects needs to be considered, also depending on the exact outcome. Moreover, there is a need to investigate the long-term effects of heartbeat perception trainings, as previous studies (Schandry and Weitkunat, 1990; Meyerholz et al., 2019; Schenk et al., 2020) have examined only short-term effects due to a single heartbeat perception training session.

Extending previous studies using a specifically designed heartbeat perception training, this study investigates both the short- and long-term effects of a 3-week heartbeat perception training on two dimensions of interoception (i.e., IAc and IS). The intervention group conducted three heartbeat perception training sessions in the laboratory (one per week) and was compared to the control group watching a neutral film. We assumed that (a) IAc and (b) IS increase due to the first heartbeat perception training (short-term effect) as well as over time (from pre- to post-measurement after 4 weeks, long-term effect) as compared to the control group.

## Materials and Methods

### Participants

Based on the location of Ulm University, Germany, participants were recruited *via* flyers, e-mais, and social media. The inclusion criteria were the following: (1) age of 18 years or above, (2) sufficient knowledge of the German language, (3) Internet access, (4) no cardiovascular diseases, (5) no heart medication or psychotropic drugs, (6) no psychotherapy during the last 12 months, and (7) no current attendance of any kind of heartbeat perception training or mindfulness-based intervention. One participant from the intervention group was excluded from the data analysis because he did not meet the inclusion criteria (refer to the abovementioned criteria). Thus, the data analysis was conducted based on a sample of *N* = 39.

### Procedure and Material

The study was conducted in accordance with the Declaration of Helsinki and approved by the local ethics committee. Initially, participants received and signed the informed consent. They were randomly assigned to the intervention (*n* = 20; 25% male participants) or to the control group (*n* = 20; 30% male participants). All participants underwent four assessments (at baseline, at each training or film session, and 1 week after the last session) in a laboratory over 4 weeks (one session per week) and filled out the online questionnaires (e.g., demographic data; Perceived Stress Scale, Cohen et al., 1983) 1 day before each laboratory session. In each of the first three sessions, the intervention group conducted a 20-min cardiac heartbeat perception training, as suggested by Meyerholz et al. (2019), whereas the control group watched a 20-min documentary film instead. At the beginning of each session, after each session, and 1 week after the last session, participants of both groups performed the heartbeat counting task (Schandry, 1981) to measure IAc and rated their confidence concerning their counted heartbeats in each interval (IS). After the participants had successfully completed all parts of the study, they received 6-course credits or 50 euros.

#### Cardiac Interoceptive Accuracy

Cardiac interoceptive accuracy was assessed using the heartbeat counting task (Schandry, 1981). The task was to focus on their own heartbeats and to count them silently during four randomized intervals (lengths: 25, 35, 45, and 60 s), without getting any information about the lengths of the intervals or any feedback about their performance quality. The participants were instructed to sit in a relaxed position, to avoid movements, and not to use manipulating strategies, such as to stop breathing and to take their pulse. Importantly, they should count solely those heartbeats they were sure of sensing. For every interval, an automated start and stop signal was presented after which the participants had to report their counted heartbeats and the confidence ratings. First, a 15-s training interval was conducted to get familiar with the task. The task instructions were presented *via* the software Presentation (Neurobehavioral Systems, version 22.1; 30 April 2021). Biopac MP150 with a sampling rate of 1,000 Hz was used for recording the heartbeats. The averaged heartbeat perception scores were calculated using the following equation:


$$IAcScore=\frac{1}{4}\sum (1-\frac{\left(\left|\mathrm{recorded}\,\mathrm{heartbeats}-\mathrm{counted}\,\mathrm{heartbeats}\right|\right)}{\mathrm{recorded}\,\mathrm{heartbeats}}$$

The score ranges from 0 to 1; higher scores indicate a higher IAc for cardiovascular signals.

#### Interoceptive Sensibility

Interoceptive sensibility (i.e., self-reported beliefs concerning IAc) was measured *via* confidence ratings, meaning that the participants had to rate how confident they felt regarding their counted heartbeats after each interval of the heartbeat counting task (Schandry, 1981). The rating scale ranged from 0 (=no confidence) to 10 (=complete confidence). The confidence items were presented *via* the software Presentation (Neurobehavioral Systems, version 22.1; 30 April 2021) and recorded.

#### Intervention: Heartbeat Perception Training vs. Control Group

**FIGURE 1:**
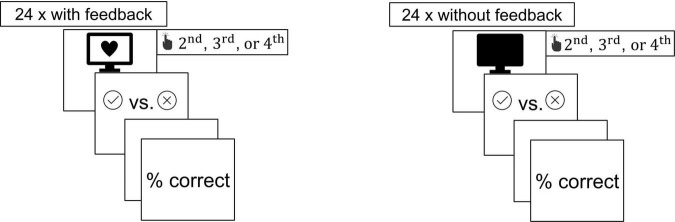
Procedure of the heartbeat perception training paradigm.

The heartbeat perception training paradigm was based on the paradigm by Schaefer et al. (2014). Participants conducted three training sessions (one per week) in a small, soundproof room within the laboratory which lasted for around 20 min. Prior to the actual heartbeat perception training, the participants underwent a phase of 15 s where only the heartbeat symbol according to the individual heartbeat was presented. Following, each training consisted of three training blocks of 48 trials, and each block was composed of two phases (24 trials per phase). In each first phase, visual biofeedback was presented 200 ms after an R-wave detection in the form of a red heart symbol. In the second phase, no such visual biofeedback was presented. In each trial of the phase, the participants were instructed to press a button when they perceived the instructed second, third, or fourth heartbeat as displayed. For this purpose, the numbers two, three, or four were pseudorandomly presented. The reaction was classified as correct if the button was pressed within 450 ms after the detection of the last heartbeat. The feedback concerning their performance, presented in all phases, consisted of a green checkmark for correct responses and a red “X” for wrong responses. At the end of each phase, the percentage of correct responses was displayed. The procedure of each heartbeat perception training session is depicted in Figure 1. Between the two phases of each block, a pause of 15 s was implemented. Twelve practice trials were followed to acquaint the participant with the procedure.

The control group watched a 20-min documentary film about architectural stylistic epochs in the laboratory instead of the heartbeat perception training. This control condition was selected as a quite neutral stimulus with an external focus only.

### Data Analysis

We reported descriptive statistics, IAc and IS mean scores, and *t*-tests for independent samples regarding demographic data and relevant variables at baseline (e.g., age, body mass index, IAc*_*pre*_*, and IS*_*pre*_*). To analyze the short-term effects of the first training session on IAc and IS, we conducted repeated-measures ANOVAs with the factors of time and group. All these analyses were calculated using the Statistical Package for Social Science (SPSS; version 27).

To investigate the effects of all three trainings over time, we used regression models. Due to the nested longitudinal data structure, we employed hierarchical linear models. The measurement points (level 1) were nested within participants (level 2). The hierarchical linear models and model comparisons were estimated in R using the packages *lme4* (Bates et al., 2014), *lmerTest* (Kuznetsova et al., 2017), and *r2mlm* (Rights and Sterba, 2019). In our regression analyses, we included the baseline measure (T1), the pre-measures of the second and the third training session (T2_*pre*_ and T3_*pre*_), and a measure that took place 1 week after the third training session (follow-up measure, T4). Thus, slopes indicate the predicted change in IAc and IS, respectively, over the course of 1 week after a training. The dichotomous predictor variable group was dummy-coded and the predictor time had an interpretable zero point (the pre-measurement before the first training). Therefore, centering the predictors group and time was not necessary. In one model, we used IAc as a predictor variable (refer to Table 3), which was grand-mean centered in this case. Due to a sample size of *N* = 39, the restricted maximum likelihood (REML) estimator was applied. To judge the model fit, the Akaike information criterion (AIC), the Bayesian information criterion (BIC), and, where appropriate, likelihood ratio tests were calculated to evaluate relative model fit. For the likelihood ratio tests, the Maximum Likelihood (ML) estimator was applied. In addition, we calculated $R_{t}^{2\,\left(f\right)}$ describing the proportion of total outcome variance explained by predictors *via* fixed slopes (Rights and Sterba, 2019, Table 5, Formula 1) and $R_{t}^{2\,\left(f\,v\,m\right)}$describing the proportion of total outcome variance explained by predictors *via* fixed and random slope variation/covariation and by person-specific outcome means (Rights and Sterba, 2019, Table 5, Formula 5). The significance level for all analyses was *p* ≤ 0.05.

**TABLE 1:** Descriptive statistics at baseline (T1) per group.

	Intervention group (*n* = 19) *Mean* (*SD*)	Control group (*n* = 20) *Mean* (*SD*)	*t*(37)	*p*
Age	25.21 (8.42)	24.90 (8.98)	–0.111	0.912
BMI	22.00 (2.15)	22.14 (2.30)	0.204	0.839
IAc*_*pre*_*	0.68 (0.22)	0.63 (0.26)	–0.630	0.533
IS*_*pre*_*	4.71 (1.75)	4.34 (1.68)	–0.679	0.501

*BMI, body mass index; IAc, cardiac interoceptive accuracy; IS, interoceptive sensibility.*

**TABLE 2:** Model 1: Random intercept and slope model for cardiac interoceptive accuracy with the predictors time, group, and the interaction of time and group.

		Model				
		β	*SE*	*df*	*t*	*p*
**Fixed effects**						
Intercept		0.623	0.049	37.001	12.685	<0.001
Level 1						
	Time	0.028	0.015	37.001	1.931	0.061
Level 2						
	Group	0.078	0.070	37.001	1.115	0.272
Cross-level-interaction						
	Time × group	0.016	0.021	37.001	0.776	0.443
		**σ** ^2^	***SD***			
**Random effects (ariance components)**						
*σ***^2^***_*u0j*_*(intercept)		0.041	0.202			
*σ*^2^*_*u01j*_*(time)		0.002	0.046			
*σ***^2^***_*rij*_*(residual)		0.011	0.104			

*β, fixed effect coefficients; σ^2^, variance of random effect coefficients; SE, standard errors; SD, standard deviations.*

**TABLE 3:** Model 5: Random intercept and random slope model for interoceptive sensibility with the predictors time, group, cardiac interoceptive accuracy, and the interaction of time and group.

		Model				
		β	*SE*	*df*	*t*	*p*
**Fixed effects**						
	Intercept	4.931	0.296	32.370	16.663	< 0.001
Level 1						
	Time	–0.028	0.118	33.324	–0.235	0.816
Level 2						
	Group	–0.377	0.411	30.966	–0.917	0.366
	IAc	3.662	0.955	21.091	3.835	< 0.001
Cross-level-interaction						
	Time × group	0.177	0.165	31.892	1.071	0.292

		**σ** ^2^	***SD***			
**Random effects (variance components)**						
σ^2^*_*u0j*_* (intercept)		0.895	0.946			
σ^2^*_*u01j*_* (time)		0.131	0.362			
σ^2^*_*u02j*_* (cardiac interoceptive accuracy)		16.523	4.065			
σ^2^*_*rij*_* (residual)		0.670	0.818			

*β, fixed effect coefficients; σ^2^, variance of random effect coefficients; SE, standard errors; SD, standard deviations.*

## Results

The relevant descriptive statistics are summarized in Table 1. There were no significant differences between the groups at baseline (T1).

### Short-Term Effect on Cardiac Interoceptive Accuracy

**FIGURE 2:**
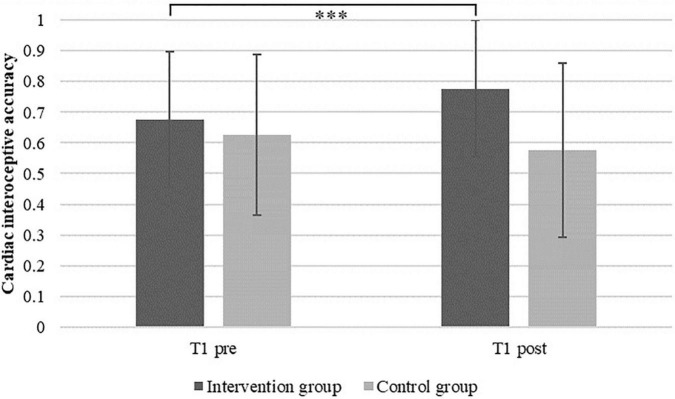
Mean cardiac interoceptive accuracy and standard errors in the intervention and the control group before (T1_*pre*_) and after (T1_*post*_) the first heartbeat perception training session (^***^p < 0.001).

First, we tested for the short-term effect after the first training. Results of the repeated-measures ANOVA revealed a significant time × group interaction effect [*F*(1, 37) = 14.268, *p* < 0.001, η*_*p*_*^2^ = 0.278], indicating an increase in IAc due to the first heartbeat perception training at T1 in the intervention group (intervention group: mean IAc_t1_*_*pre*_* = 0.676, *SE* = 0.222; mean IAc_t1_*_*post*_* = 0.777, *SD* = 0.224; control group: IAc_t1_*_*pre*_* = 0.627, *SD* = 0.262; IAc_t1_*_*post*_* = 0.577; *SD* = 0.285). The main effects of time [*F*(1, 37) = 1.587, *p* = 0.216, η*_*p*_*^2^ = 0.041] and group [*F*(1, 37) = 2.584, *p* = 0.116, η*_*p*_*^2^ = 0.065] were not significant. Mean values and standard errors are depicted in Figure 2.

### Short-Term Effect on Interoceptive Sensibility

Regarding IS, descriptive data indicated an increase in IS due to the first heartbeat perception training at T1 in the intervention group (mean IS_t1_*_*pre*_* = 4.711, *SD* = 1.755; mean IS_t1_*_*post*_* = 4.829, *SD* = 1.581) as compared to a descriptive decrease in the control group (IS_*t*1_*_*pre*_* = 4.338, *SD* = 1.677; IS_*t*1_*_*post*_* = 4.025; *SE* = 2.071). Nevertheless, the results of the repeated-measures ANOVA revealed neither a significant effect of time [*F*(1, 37) = 0.211; *p* = 0.648; η*_*p*_*^2^ = 0.006], nor of group [*F*(1, 37) = 1.229; *p* = 0.275; η*_*p*_*^2^ = 0.032] or the time × group interaction [*F*(1, 37) = 1.041; *p* = 0.314; η*_*p*_*^2^ = 0.027].

### Effect on Cardiac Interoceptive Accuracy Over Time

**FIGURE 3:**
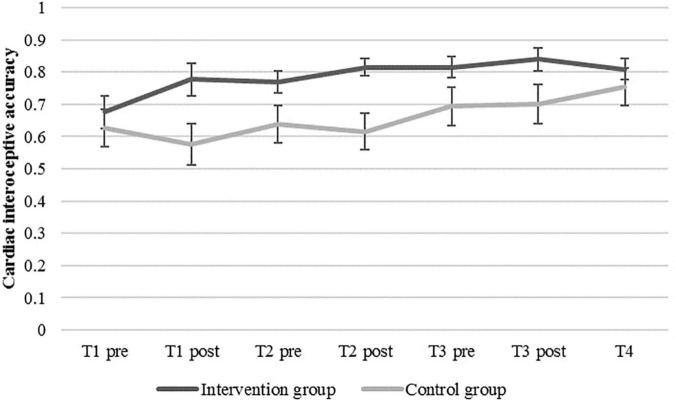
Mean cardiac interoceptive accuracy and standard errors in the intervention and the control group at baseline (T1_*pre*_), before and after each of the three heartbeat perception training sessions (pre vs. post), and post-measurement (T4).

Turning our attention from the effects of the first (single) training to the effects of all three trainings over time, the descriptive data revealed the trends of improved IAc in both groups beginning from T1 (refer to means in Figure 3). Moreover, besides the significant increase in IAc from T1_*pre*_ to T1_*post*_, on the descriptive level, results showed slight increases in IAc from T1_*pre*_ to T2_*pre*_ and from T2_*pre*_ to T3_*pre*_ in the intervention group, whereas the control group exhibited slight increases in IAc over time from T2_*pre*_ to T3_*pre*_ and from T3_*pre*_ to T4.

To test our hypothesis on the effects of repeated trainings, we predicted IAc by time, group, and the interaction of both (Model 1). In the models, four measurement points were included, namely, the baseline measure (T1), the pre-measures of the second and the third training session (T2_*pre*_ and T3_*pre*_), and a measure that took place 1 week after the third training session (follow-up measure, T4). Since repeated trainings are often accompanied by saturation effects that cannot be adequately described by linear equations, we estimated two models, one of which additionally included a quadratic effect for time (Model 2). Model 1 showed lower, i.e., better information criteria (AIC = −120.50, BIC = −96.098) than Model 2 (AIC = −120.94, BIC = −93.493). The likelihood ratio test did not show a significant difference of the deviances [deviance*_*M1*_* = −136.50; deviance*_*M2*_* = −138.94, χ^2^(1) = 2.445, *p* = 0.118]. The proportion of variance explained by the fixed slopes, $R_{t}^{2\,\left(f\right)}$, was 0.08 for Model 1 and similarly for Model 2. The variance explained by fixed slopes, random slope variation/covariation, and person-specific IAc means, $R_{t}^{2\,\left(f\,v\,m\right)}$, was 0.80 for both models. Thus, Model 1 was chosen for our regression analysis regarding IAc.

Table 2 shows the results of this random intercept and random slope model (Model 1). According to this model, the significant fixed effect of the intercept indicates an estimated mean IAc of *β_00_* = 0.623 (*SE* = 0.049; *p* < 0.001) in the control group (i.e., the reference group) before the first training (T1*_*pre*_*). The variance of intercepts σ^2^*_*u*0j_* = 0.041 (*SD* = 0.202) describes the heterogeneity in participants’ IAc scores at T1*_*pre.*_* The fixed effect of the level-1-predictor time (*β_01_* = 0.028; *SE* = 0.015; *p* = 0.061) was not significant, indicating that the factor of time did not predict IAc. Random effects for this coefficient indicate large differences between participants in growth over time (σ^2^*_*u0*1j_* = 0.002, *SD* = 0.046). The fixed effect of the level-2-predictor group was not significant (*β_02_* = 0.078; *SE* = 0.070; *p* = 0.272), indicating no significant differences in IAc between the groups. Furthermore, the cross-level interaction of the variables time and group (*β_03_* = 0.016; *SE* = 0.021; *p* = 0.443) was not significant. Thus, the increase in IAc was not stronger in the intervention group than in the control group.

### Effect on Interoceptive Sensibility Over Time

**FIGURE 4:**
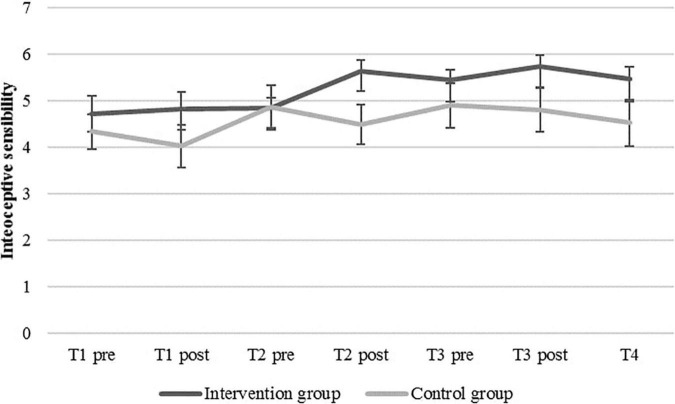
Mean confidence levels (interoceptive sensibility) and standard errors in the intervention and the control group at baseline (T1_*pre*_), before and after each of the three heartbeat perception training sessions (pre vs. post), and post-measurement (T4).

Figure 4 shows the means for IS (i.e., the confidence ratings) over time. There are slight descriptive trends of increased IS in both groups, with a slight decrease in the control group to T4. Again, we first selected an appropriate model for the data. We compared a model that used time, group, and the interaction of time and group (Model 3), a model that used the same predictors plus a quadratic term for time to model potential non-linear effects (Model 4), and a model without quadratic effects but with IAc as an additional predictor (Model 5). Model 5 showed lower (i.e., better) information criteria (AIC = 521.59, BIC = 558.18) than Model 3 (AIC = 555.40, BIC = 579.80) and Model 4 (AIC = 554.68, BIC = 582.13). The proportion of variance explained by the fixed slopes, $R_{t}^{2\,\left(f\right)}$, was 0.04 for Model 0.04 for Model 4, and 0.22 for Model 5. The variance explained by fixed slopes, random slope variation/covariation, and person-specific IS means, $R_{t}^{2\,\left(f\,v\,m\right)}$, was 0.67 for Model 0.068 for Model 4, and 0.80 for Model 5. Accordingly, the results of the likelihood ratio test comparing Model 3 and Model 5 showed a significant better fit of Model 5 [deviance*_*M3*_* = 539.40; deviance*_*M5*_* = 497.59; χ^2^(4) = 41.818, *p* < 0.001].

Table 3 shows the results for this model with a random intercept and random slopes for the predictors of time and group, the interaction of time and group, and IAc (Model 5). The intercept of *β_00_* = 4.931 (*SE* = 0.296; *p* < 0.001) is the predicted IS for a control group participant with an average IAc at the first measurement point (T1_*pre*_). The variance of random intercepts (σ^2^*_*u*0j_* = 0.895; *SD* = 0.946) represents heterogeneity of IS levels at the beginning of the study (T1*_*pre*_*). The non-significant fixed effect of the level-1-predictor time (*β_01_* = −0.28; *SE* = 0.118; *p* = 0.816) shows that IS did not significantly increase over the course of time. The fixed effect of the level-2-predictor group was also not significant (*β_02_* = −0.377; *SE* = 0.411; *p* = 0.366), indicating no significant differences in IS between the groups. The significant fixed effect of the level-1-predictor IAc (*β_03_* = 3.662; *SE* = 0.955; *p* < 0.001) indicates that higher levels of IAc are associated with higher levels of IS. Furthermore, the cross-level-interaction of the variables time and group (*β_03_* = 0.177; *SE* = 0.165; *p* = 0.292) was not significant. Thus, there was no superior change in IS in the intervention group compared with the control group.

## Discussion

The aim of this study was to examine the effects of a 3-week heartbeat perception training on interoceptive abilities in a randomized controlled trial. After the first training session, IAc was significantly increased in the intervention group, indicating a short-term effect on IAc. There was no significant short-term effect on IS due to the first training session. Regarding the long-term effects, there was neither a significant effect on IAc nor on IS.

### Short-Term Effect on Cardiac Interoceptive Accuracy

As we hypothesized, IAc increased significantly due to the first session of the heartbeat perception training in the intervention group with higher effects as compared to the control group. This short-term effect is in line with the results by Meyerholz et al. (2019) and Schenk et al. (2020). In contrast, in the study by Schaefer et al. (2014) and in the recent replication study by Rominger et al. (2021), no short-term effect on IAc could be found. Schaefer et al. (2014), Meyerholz et al. (2019), and Schenk et al. (2020) as well as the present study used the same cardiac heartbeat perception training paradigm by Schaefer et al. (2014) and the heartbeat counting task (Schandry, 1981) to quantify IAc. Whereas Schaefer et al. (2014) tested a clinical sample and 17 conducted a second heartbeat counting task after the training session 14 days later, in the studies 18 by Meyerholz et al. (2019) and Schenk et al. (2020) as well as in our study, healthy samples were 19 investigated and IAc was assessed immediately before and after the training (short-term effect). In this context, it should be emphasized that Rominger et al. (2021) used the heartbeat discrimination task (Whitehead et al., 1977) to assess IAc directly before and after the heartbeat perception training paradigm by Meyerholz et al. (2019), comparing the effects with a breathing intervention and a film condition. As the tasks differ in their focus of attention, namely, both an external and an internal focus on the heartbeat discrimination task vs. an exclusive focus on internal signals in the heartbeat counting task (Schulz et al., 2013; Couto et al., 2015; Ring and Brener, 2018), it is possible that these inconsistent findings result from methodological differences. These differences exist in the measurement instrument, the investigated sample, and the time of measurement.

In line with the explanatory approaches by Meyerholz et al. (2019) and Schenk et al. (2020), our results could be explained by an immediate interoceptive learning effect due to the heartbeat perception training. Moreover, we assumed that the training evoked a high internal focus of attention, especially in the training phases without the performance-related feedback. This focus might have supported the individual heartbeat perception in contrast to an external focus on the control condition generated by the neutral film. Complementing this approach, Petzschner et al. (2019) showed that the heartbeat-evoked potential, an index of the cortical processing of cardiac interoceptive signals (e.g., Pollatos and Schandry, 2004; Mai et al., 2018), is modulated by attention, as the heartbeat-evoked potential was higher during interoceptive compared to exteroceptive attention.

Explaining the interoceptive learning effect within the framework of predictive coding (e.g., Seth et al., 2012; Friston et al., 2014; Allen and Tsakiris, 2019; Petzschner et al., 2021), perceptual learning, based on cardiac stimuli, individual IAc and IS could have resulted in the minimization of prediction errors (i.e., discrepancies between current and previous individual perceptions). Integrating the approaches proposed by Canales-Johnson et al. (2015) and Hodossy and Tsakiris (2020), reduced prediction errors could have induced updated predictive models of interoceptive signals including changes in priors (i.e., individual models of perception), presumably on the perception of cardiac signals, representing a learning effect. Canales-Johnson et al. (2015) showed that cardiac interoceptive learning based on auditory feedback is associated with changes in heartbeat-evoked potential amplitudes. Furthermore, they found evidence for a network hub in the insular cortex for cardiac interoceptive learning due to a heartbeat-tapping task and auditory feedback. In addition, Schenk et al. (2020) also suggested that the anterior insula and the anterior cingulate cortex, which were identified as central for IAc (e.g., Pollatos et al., 2005a,b), might play a pivotal role in interoceptive learning and might account for the neurophysiological base.

### Short-Term Effect on Interoceptive Sensibility

Contrarily to our hypothesis, IS did not increase after the first heartbeat perception training session. It needs to be noted that our study is the first that investigated IS in the context of a heartbeat perception training. Similarly, Weineck et al. (2020b) found increased IAc due to a single session of power posing in healthy women, whereas there was also no change in IS as assessed *via* confidence ratings and *via* the Body Perception Questionnaire (Porges, 1993, 2015). The authors argue that interventions targeting individuals’ self-focus might not be directly associated with increases in performance confidence. Transferring the approach by Weineck et al. (2020b), the heartbeat perception training might have evoked a high internal focus of attention (i.e., self-focus) and might account for the missing effect on IS due to a single session of heartbeat perception training. Moreover, the performance feedback in the heartbeat perception training could have had an impact on the perceived confidence regarding IAc, for instance, by unsettling the participants in their confidence due to the feedback if their response was incorrect. Finally, this result is in accordance with other studies (Garfinkel et al., 2015; Forkmann et al., 2016; Meessen et al., 2016; Murphy et al., 2019; Weineck et al., 2019), showing that IAc and IS are distinct dimensions of interoception that might differentially benefit from various interventions.

### Effect on Cardiac Interoceptive Accuracy Over Time

Contrary to our hypothesis, there was no significant increase in IAc over the course of the three trainings. Descriptive trends of improved IAc were found in both groups. Similarly, Schaefer et al. (2014) reported increased IAc in the waiting control group after 2 weeks and explained this result by a time effect or a learning effect from the heartbeat counting task itself, which might also account for the present findings.

More importantly, Weineck et al. (2020b) found only a short-term effect on IAc due to a single session of power posing as an intervention aiming to improve interoceptive abilities, but no effect after 1 week. Central reasons for the missing effects could be that the duration or the intensity (number of sessions) of the intervention was not sufficient to improve IAc. In particular, previous research on body-oriented interventions aiming to improve interoceptive abilities mostly showed effects after longer interventions of at least 8 weeks (Bornemann et al., 2014; Bornemann and Singer, 2017; Fischer et al., 2017).

A further contributing factor of interest could be that the whole heartbeat perception training was too unspecific to influence IAc over time, as the focus is on different body sensations as discussed in the context of mindfulness-based interventions (Khalsa et al., 2008; Meyerholz et al., 2019).

### Effect on Interoceptive Sensibility Over Time

In contrast to our hypothesis, IS did not increase significantly over the course of the intervention. Contrarily to the present findings, Fischer et al. (2017) showed significantly higher confidence due to an 8-week mindfulness-based intervention in both the intervention and the active control group. Moreover, Parkin et al. (2014) found increased confidence due to an 8-week mindfulness-based intervention. As compared to the previous findings regarding IS in studies with mindfulness-based or self-focus-related interventions (Parkin et al., 2014; Fischer et al., 2017; Weineck et al., 2019) and similar to the results concerning IAc over time, the missing effects could be explained by an insufficiently low duration or intensity of the intervention. Furthermore, as discussed regarding the short-term effect on IS, feedback on the performance in the heartbeat perception training could have had an impact on the perceived confidence regarding IAc. Future studies should control for this aspect by explicitly instructing the participant that the confidence levels concerning IAc have to be rated independently from the performance in the biofeedback paradigm.

Importantly, although there was no significant change in IS, the present results indicate that IAc is associated with IS. This is in line with the findings by Garfinkel et al. (2015) who report an association between IAc assessed *via* the heartbeat counting task and IS quantified *via* confidence ratings. However, the present result needs to be interpreted carefully, as IS was not directly manipulated in our experiment and did not change significantly over time. Thus, the variance of IS explained by IAc is likely between-person variance, that is, even though IS was constant or did not systematically covary with time or group membership, participants with higher levels of IAc also showed higher levels of IAc independently from our experimental manipulations. Nonetheless, IAc and IS need to be considered distinct facets as shown in previous studies (e.g., Garfinkel et al., 2015; Forkmann et al., 2016; Weineck et al., 2019).

### Strengths, Limitations, and Future Research

A strength of this study is that both short-term effects and the effects over the time of a 3-week heartbeat perception training on health-related interoceptive abilities were investigated. Furthermore, by calculating hierarchical linear models, intraindividual and interindividual differences in the outcome variables were considered. Moreover, in contrast to previous studies, two dimensions of interoception, namely, IAc and IS, were assessed. In addition, we replicated the short-term effect on IAc of the study by Meyerholz et al. (2019), which highlights the effectiveness of single heartbeat perception training sessions aiming to improve IAc. In addition, examining the effects of several heartbeat perception training sessions on interoceptive abilities can be underlined as an innovative approach.

Concerning the debate about the heartbeat counting task including criticism (e.g., Ring et al., 2015; Murphy et al., 2018; Ring and Brener, 2018; Zamariola et al., 2018) and contrary approaches regarding the criticism (Ainley et al., 2020; Zimprich et al., 2020), it can be mentioned that the strict instruction (i.e., to count exclusively those heartbeats of which the participants actually perceived) reduces the risk that the knowledge about the individual heartbeat influences IAc (Schulz and Vögele, 2015). It might be important to shed light on the type of instructions applied in the heartbeat counting task. In this study, the strict instruction was used. For example, in similar intervention studies aiming to improve IAc in healthy samples, mean IAc scores, assessed pre-intervention, are comparable with the IAc mean scores found in this study. These mean scores were reported between 0.56 and 0.70 (Fischer et al., 2017; Meyerholz et al., 2019; Weineck et al., 2019; Schenk et al., 2020). Furthermore, it needs to be noted that in previous research using the heartbeat counting task (e.g., Kever et al., 2015; Fischer et al., 2017; Meyerholz et al., 2019), commonly, information about the exact instruction (strict vs. standard) were not reported. In contrast and comparably to our study, Schenk et al. (2020) described similar IAc mean scores as compared to our reported scores by using the strict instruction of the heartbeat counting task. Desmedt et al. (2018) reported significantly lower mean IAc scores due to the strict instruction of the heartbeat counting task. Nevertheless, the study design in terms of conducting the (adapted) heartbeat counting task differed as compared to other studies (e.g., Fischer et al., 2017; Meyerholz et al., 2019; Weineck et al., 2019; Schenk et al., 2020). Due to diverse instructions and a lack of information on the detailed instructions in previous research, comparing the mean scores in IAc might be difficult. Consequently, the type of instruction might be a potential influencing factor on IAc.

Pointing out the limitations, first, the study investigated a non-clinical sample that was not characterized by low interoceptive abilities. Therefore, future research should examine diverse samples, especially those with low interoceptive abilities, such as clinical populations or samples with high-stress levels aiming to improve interoceptive abilities as related to health benefits, such as better regulation of emotions (e.g., Barrett et al., 2004; Herbert and Pollatos, 2008; Dunn et al., 2010; Füstös et al., 2013; Zamariola et al., 2019a), or a better perception of bodily symptoms (van den Bergh et al., 2019). Moreover, future studies should design interventions over a longer period, implement a higher frequency of sessions, or include psychoeducative elements. Integrating training elements into the participants’ everyday life might be important to demonstrate the practical health relevance of improving interoceptive abilities. Therefore, further training elements could be smartphone-based biofeedback (Dillon et al., 2016) or portable biofeedback devices (Lemaire et al., 2011). A possible approach for future training to improve interoceptive abilities might be a combination of training sessions in the laboratory and *via* portable devices or smartphones to better integrate the training into everyday life. In addition, further extensions could be innovative smartphone-based heartbeat counting tasks (Plans et al., 2021) or breathing-related perception training or tasks, such as the filter detection task (Harrison et al., 2021), to focus on another interoceptive dimension. According to the 2 × 2 factorial model of interoceptive abilities by Murphy et al. (2019), distinguishing between accuracy and attention to interoceptive signals (i.e., factor 1) and between objective measures and self-reported beliefs concerning interoceptive signals (i.e., factor 2) is essential. In line with the model by Murphy et al. (2019), ecological momentary assessments might be used to assess interoceptive states in everyday life (Bornemann and Singer, 2017; Kok and Singer, 2017) and to sensitize for a more frequent attention to interoceptive signals, i.e., potentially, not only focusing on cardiac but also respiratory or gastrointestinal signals. Furthermore, in addition to confidence ratings, appropriate questionnaires, such as the Interoceptive Accuracy Scale (Murphy et al., 2019) or the Interoceptive Confusion Questionnaire (Murphy et al., 2019), to quantify IS. Finally, the study should be replicated based on a larger sample to increase the power.

To sum up, a single session of a cardiac heartbeat perception training seems to be a promising approach to improve IAc. A heartbeat perception training over 3 weeks might be insufficient to improve interoceptive abilities from pre- to post-intervention. Future research should further investigate the effects of various heartbeat perception trainings over at least 3 weeks or longer, varying in frequency and intensity of the training sessions and, potentially, complementary mobile elements in diverse samples aiming to improve interoceptive abilities. Furthermore, heartbeat perception training could be a promising approach for clinical samples.

## Data Availability Statement

The original contributions presented in the study are included in the article/Supplementary Material, further inquiries can be directed to the corresponding author.

## Ethics Statement

The studies involving human participants were reviewed and approved by the Ethics Commitee of Ulm University. The patients/participants provided their written informed consent to participate in this study.

## Author Contributions

CS, GK, and OP designed the study. CS and GK conducted the study. CS and NS analyzed the data. CS wrote the first draft of the manuscript. GK, DS, and OP edited the manuscript. All authors discussed the results and approved the final version of the manuscript for submission.

## Conflict of Interest

The authors declare that the research was conducted in the absence of any commercial or financial relationships that could be construed as a potential conflict of interest.

## Publisher’s Note

All claims expressed in this article are solely those of the authors and do not necessarily represent those of their affiliated organizations, or those of the publisher, the editors and the reviewers. Any product that may be evaluated in this article, or claim that may be made by its manufacturer, is not guaranteed or endorsed by the publisher.
