# Supplementary material for: The Effects of a 3-Week Heartbeat Perception Training on Interoceptive Abilities
Source: Front Neurosci. 2022 May 9;16:838055. doi: 10.3389/fnins.2022.838055 (PMC9124832; doi:10.3389/fnins.2022.838055)
Supplement: Supplementary file 1 [file Data_Sheet_1.docx]

**Supplementary Material**

**Appendix**

**A1.** *Model 2****:*** *Random intercept and random slope model for cardiac interoceptive accuracy with the predictors time, the quadratic effect of time, group, and the interaction of time and group.*

|  |  |  |  |  | Model |  |  |
| --- | --- | --- | --- | --- | --- | --- | --- |
|  |  |  | *β* | *SE* | *df* | *t* | *p* |
| **Fixed effects** |  |  |  |  |  |  |  |
|  | Intercept |  | .610 | .050 | 39.113 | 12.249 | < .001 |
|  | Level 1 |  |  |  |  |  |  |
|  |  | Time | .067 | .029 | 111.808 | 2.331 | .022 |
|  | Level 2 |  |  |  |  |  |  |
|  |  | Group | .078 | .070 | 37.000 | 1.115 | .272 |
|  | Interaction |  |  |  |  |  |  |
|  |  | Time * time | -.013 | .008 | 77.000 | -1.566 | .122 |
|  | Cross-level-interaction |  |  |  |  |  |  |
|  |  | Time * group | .016 | .021 | 37.000 | .776 | .443 |
|  |  |  |  |  |  |  |  |
|  |  |  | *σ²* | *SD* |  |  |  |
| **Random effects (Variance components)** |  |  |  |  |  |  |  |
|  | *σ²_u0j_*  (Intercept) |  | .041 | .202 |  |  |  |
|  | *σ²_u01j_* (Time) |  | 0.002 | .047 |  |  |  |
|  | *σ²_rij_* (Residual) |  | .011 | .103 |  |  |  |

*β* = fixed effect coefficients; *σ² =* random effect coefficients; *SE* = standard errors; *SD* = standard deviations; *p* = p-values.

**A2*.*** *Model 3:* *Random intercept and random slope model for interoceptive sensibility with the predictors time, group, and the interaction of time and group.*

|  |  |  |  |  | Model |  |  |
| --- | --- | --- | --- | --- | --- | --- | --- |
|  |  |  | *β* | *SE* | *df* | *t* | *p* |
| **Fixed effects** |  |  |  |  |  |  |  |
|  | Intercept |  | 4.571 | .357 | 36.999 | 12.816 | < .001 |
|  | Level 1 |  |  |  |  |  |  |
|  |  | Time | .059 | .132 | 37.000 | 0.443 | .660 |
|  | Level 2 |  |  |  |  |  |  |
|  |  | Group | .113 | .511 | 36.999 | .221 | .826 |
|  | Cross-level-interaction |  |  |  |  |  |  |
|  |  | Time* group | .231 | .190 | 37.000 | 1.214 | .232 |
|  |  |  |  |  |  |  |  |
|  |  |  | *σ²* | *SD* |  |  |  |
| **Random effects (Variance components)** |  |  |  |  |  |  |  |
|  | *σ²_u0j_*  (Intercept) |  | 1.858 | 1.363 |  |  |  |
|  | *σ²_u01j_*  (Time)  *σ²_rij_* (Residual) |  | .156  .981 | .395  .990 |  |  |  |

*β* = fixed effect coefficients; *σ² =* random effect coefficients; *SE* = standard errors; *SD* = standard deviations; *p* = p-values.

**A3.** *Model 4:* *Random intercept and random slope model for interoceptive sensibility with the predictors time and group, the quadratic effect of time, the interaction of time and group.*

|  |  |  |  |  | Model |  |  |
| --- | --- | --- | --- | --- | --- | --- | --- |
|  |  |  | *β* | *SE* | *df* | *t* | *p* |
| Fixed effects |  |  |  |  |  |  |  |
|  | Intercept |  | 4.441 | .365 | 40.617 | 12.162 | < .001 |
|  | Level 1 |  |  |  |  |  |  |
|  |  | Time | .448 | .270 | 110.501 | 1.659 | .100 |
|  | Level 2 |  |  |  |  |  |  |
|  |  | Group | .113 | .511 | 36.999 | .221 | .826 |
|  | Interaction |  |  |  |  |  |  |
|  |  | Time* time | -.130 | .078 | 77.001 | -1.655 | .102 |
|  | Cross-level-interaction |  |  |  |  |  |  |
|  |  | Time* group | .231 | .190 | 36.999 | 1.214 | .233 |
|  |  |  |  |  |  |  |  |
|  |  |  | *σ²* | *SD* |  |  |  |
| Random effects (Variance components) |  |  |  |  |  |  |  |
|  | *σ²_u0j_*  (Intercept) |  | 1.873 | 1.369 |  |  |  |
|  | *σ²_u01j_* (Time)  *σ²_rij_* (Residual) |  | .160  .959 | .400  .976 |  |  |  |

*β* = fixed effect coefficients; *σ²* = random effect coefficients; *SE* = standard errors; *SD* = standard deviations; *p* = p-values.
